# Health-related quality of life among patients with bipolar disorder in rural southwestern Uganda: a hospital based cross sectional study

**DOI:** 10.1186/s12955-021-01729-5

**Published:** 2021-03-10

**Authors:** Lucas Anyayo, Scholastic Ashaba, Mark Mohan Kaggwa, Samuel Maling, Etheldreda Nakimuli-Mpungu

**Affiliations:** 1 Lira University, Lira, Uganda; 2 Mbarara University of Science and Technology, Mbarara, Uganda; 3 Makerere University College of Health Sciences, Kampala, Uganda

**Keywords:** Bipolar disorder, Remission, Quality of life, Uganda

## Abstract

**Background:**

Bipolar disorder is a psychiatric disorder that alters mood and affects over 55 million people globally with an estimated lifetime prevalence of approximately 0.8–1.1%. In Africa, the lifetime prevalence of the bipolar spectrum disorders is slightly lower at 0.1–0.6%. Bipolar disorder is ranked the sixth leading cause of disability with high rates of morbidity and mortality and negatively impacts quality of life of those affected.

**Methods:**

The aim of the study was to determine the health-related quality of life of patients with bipolar disorder attending a mental health clinic in south western Uganda. We enrolled a consecutive sample of 169 participants and evaluated their health-related quality of life using the medical outcomes health survey short form-36 (SF-36) scale. We used bivariate and multivariable logistic regression to determine associations between quality of life, sociodemographic and clinical factors setting the physical and mental component categories of quality life scale as the main outcome variables.

**Results:**

The mean age of the participants was 37.23 (12.83) and slightly over half (54.4%) were females. More than half (66.86%) of the participants had poor physical component summary (mean = 45.06, SD = 8.44) while 81% of the participants had poor mental component summary (mean = 41.95, SD = 8.45). Poor physical quality of life had a statistically significant association with history of suicidal thoughts (OR = 2.75, 95% CI = 1.14–6.63, *P* = 0.02), while poor mental quality of life had a statistically significant association with history of suicidal thoughts (OR = 3.94, CI = 1.22–12.71, *P* = 0.02) and history of psychotic symptoms (OR = 2.46, CI = 1.07–5.64, *P* = 0.03).

**Conclusion:**

The mental and physical quality of life of our participants was poor and history of suicidal thoughts and psychotic symptoms were associated with poor quality of life. There is need to address psychotic symptoms and suicidal thoughts in the management of patients with bipolar disorder to improve health related outcomes and quality of life.

## Background

Bipolar disorder (BD) is a mental illness marked by extreme shifts in mood ranging from manic to depressive states [1, 2]. Bipolar disorder is the 6th leading cause of disability [3] among the non-communicable diseases and various studies estimate the prevalence to be between 1 and 2% [4, 5]. BD affects over 54 million people worldwide [6] and is associated with high levels of morbidity and mortality [7]. BD has an estimated 4.4–10.3 (7%) disability-adjusted life years (DALYs), making it the 7th leading cause of years of life lost (YLL) and years lived with disability (YLD) [8]. In Africa, the lifetime prevalence of bipolar spectrum disorders based on surveys done in Egypt and Nigeria was found to be between 0.1 and 0.6% [9]. Bipolar disorder has a chronic course characterized by frequent and recurrent episodes and this chronicity causes significant impairment in functioning [10–13] and a considerable amount of disability even after remission of symptoms [14–16]. Estimates show that 25–50% of patients with bipolar disorder attempt suicide in their lifetime while about 15–19% commit suicide [17].

Previous studies show that the quality of life (QoL) of patients with BD is poorer compared to the general population [18, 19]. According to the World Health Organization (WHO), quality of life is a constellation of different factors in relation to an individual and their environment including how they perceive their position in society in the context of their culture, their goals, expectations and concerns [20]. Health related quality of life comprises of different aspects of life including psychological, social and physical functioning and improved health related QoL is a good indicator of improved functioning for people with bipolar disorder and other chronic conditions [21, 22]. Presence of depressive symptoms and illicit drug use among people with bipolar disorder are associated with worsened quality of life [23, 24]. Additionally presence of psychotic symptoms during a manic episode is associated with slow recovery and high relapse rates which affect functionality [25, 26]. People with bipolar disorder experience compromised quality of life with profound impact on different domains including education, work productivity and intimate relationships [21, 27]. Impaired quality of life has been reported to persist even when patients are in remission [28–30]. At individual level, there is a significant alteration of one's cognition, sleeping patterns, impairment in occupational functioning and disturbance in interactions with family and friends [31, 32].

BD negatively impacts health-seeking behaviors and the treatment outcomes which in turn affects the individuals' ability to function properly in society [33, 34]. Moreover, when bipolar disorder starts early in life it causes poorer global functioning, higher rates of academic failure, poor interpersonal relationships, and a high risk of suicide attempts [35, 36]. There is a high rate of marital dysfunction in patients with BD, including hyper and hypo-sexuality, promiscuity, lack of self-control and misuse of family assets, and as a result, the rate of divorce is high among couples where a spouse has bipolar disorder [37]. Divorce and separation are more common among people with bipolar disorder than in the general population [38–40]. Furthermore, BD is highly comorbid with other medical conditions like cardiovascular diseases, HIV and other psychiatric disorders like anxiety disorders, alcohol and drug abuse [41, 42], which further affect the QoL of the affected individuals.

Several factors have been shown to contribute to the decline in functioning among people with bipolar disorder including lack of social support and poor health-seeking behaviour [29, 43]. In sub-Sahara Africa, the quality of life of patients with bipolar disorder may be worse given the numerous challenges encountered in the management and support of individuals with mental illness [44, 45]. For example, in Uganda, with a population of approximately 40 million people [46], there are less than 30 psychiatrists, meaning that for every 1.3 million people, there is one psychiatrist. The country’s economic situation and social support system, which leaves many people unemployed with minimal education, causes a further decrement in the quality of life of people with bipolar disorder [47]. Despite the negative impact that BD has on the social and psychological functioning of the affected individuals and their QoL, the health-related quality of life of patients with bipolar disorder in Uganda is not well established. The aim of this study was to determine the health-related quality of life and the factors associated among patients with bipolar disorder in rural southwestern Uganda.

## Materials and methods

### Study area and study participants

We collected data between April 15 and June 20, 2017 at the outpatient mental health clinic of Mbarara Regional Referral Hospital (MRRH) in Mbarara District MRRH is a public health facility in southwestern Uganda located 270 km from Kampala, the capital city. We recruited patients with a diagnosis of bipolar disorder (as confirmed by the attending clinicians) who were in remission phase, 18 years and older, both male and female, and were attending the outpatient mental health clinic at MRRH during the study period. We excluded participants who presented with active symptoms of mental illness that would interview with their cognitive ability to understand the contents of the questionnaire and the consent documents and those who were physically unwell to stand the length of the interview at the time of recruitment.

### Sample size estimation

We used an online OpenEpi software, http://www.openepi.com, based on Kelsey and colleagues [48] to calculate the sample size. Using this formula, we used a confidence level of 95%, power of 80% and ratio of the sample size of the healthy population to that of participants with bipolar disorder to be one. We assumed that the percentage of the healthy population with a poor quality of life to be 20 and the percentage of patients with bipolar disorder with poor quality to be 40. Substituting all the numbers we arrived at sample size of 169 participants. We recruited participants consecutively as they came to the clinic for review every week until we reached our desired sample size.

### Measures

We used a locally generated questionnaire to collect  social demographic and clinical information of the participants including age, sex, marital status, level of education and source of income. Information on clinical factors included age at onset of the illness, number of episodes in the past year, number of hospital admissions in the past year and presence of suicidal thoughts and psychotic symptoms in the last acute episode. We assessed health related quality of life using the medical outcomes health survey short form-36 (SF-36) scale [49].The SF-36 is a 36-item, self-administered measure of QoL that was developed to examine the impact of disease on perceived well-being. It consists of eight subscales that measure different areas of functioning including: (1) physical functioning; (2) role disruption because of physical difficulties (role disruption-physical); (3) role disruption caused by emotional difficulties (role disruption-emotional); (4) social functioning; (5) mental health; (6) vitality; (7) general health; and (8) bodily pain. Each item is scored on 6 point Likert-type scale. Four of these domains are considered to relate to mental health, namely: (1) role limitations due to personal or emotional problems (“role-emotional”); (2) emotional wellbeing, (3) social functioning and (4) energy and fatigue. Together, these four scores form a single mental component scale, while the other four sub-scales (physical functioning, role disruption because of physical difficulties, vitality and general health) aggregate into a physical component scale. Physical (PCS) and mental (MCS) component summary measures were calculated by weighting each SF-36 item using a norm-based scoring method given in the guidelines [50, 51]. Since Uganda does not have a population norm, the norm used was that of the Tanzania population while adapting the SF 36 [52]. Pre-coded numeric values from the questionnaire, on a Likert type scale, were recorded according to the scoring key given and items were then scored in such a way that the higher the score, the better the health state [51]. Each item was scored on a 0–100 range with 0 being the lowest and 100 the highest possible score. The summary statistics (i.e. Physical and Mental Summary Scores, PCS and MCS respectively) were calculated by averaging the different normalized scales. Finally, the summary scores were then standardized to give a mean of 50 and a standard deviation of 10 by multiplying the PCS and MCS scores by 10 and adding 50 to the product. Higher scores indicate better health on each of the sub-scales [22, 51, 53].

The tool has been used in Uganda and other East Africa countries with good reliability measures [52, 54, 55]. The tool was translated into Runyankore, (the local language in southwestern Uganda), using recommended guidelines (Maxwell, 1996; Peters and Passchier, 2006; Gudmundsson, 2009). We adjusted the SF-36 to fit the context of the study setting. Several phrases and actions in the physical activity section (i.e. climbing a flight of stairs, playing golf, walking one block, walking several blocks and pushing a vacuum cleaner) were replaced with appropriate activities that suit the local context. For example, climbing a flight of stairs was changed to climbing a hill, pushing a vacuum cleaner changed to lifting a 20-L can of water. The adaptations were made following the Swahili version of the SF-36 that was adapted for use in Tanzania [52].

## Ethics

The study was approved by the Research Ethics Committee (REC) of Mbarara University of Science and Technology (#19/11–16) and Uganda National Council for Science and Technology (# SS4309). All study participants provided written informed consent and received no facilitation for participation in the study. The data collected were confidential and anonymous with no information linking the study participants to the data. Participants who got severe distress during the interview were referred to a counsellor for appropriate care.

### Data analysis

We summarized categorical data as frequencies and percentages while continuous data were summarized as means and standard deviation (SD). We carried out a bivariate analysis using logistic regression for all the predictor variables and the outcome variables. The outcome variables were the physical and mental component categories of the quality of life scale.

Quality of life summary scores (i.e. physical (PCS) and mental (MCS) summary Scores) were calculated by averaging the different normalized scales from the relevant subscales. Values below 50 indicated poor health-related quality of life and those above 50 indicated a good quality of life [50, 51]. We recorded unadjusted odds ratios, *p *values and confidence intervals and summarized findings in tables. We controlled for sex and age to determine adjusted odds, at 95% confidence intervals and statistical significance was determined at a *p *value of < 0.05. We then ran a multivariable logistic regression analysis with all the independent variables by including in the model all factors that had a *p *value of ≤ 0.3 at bivariate analysis.

## Results

Of the 169 participants enrolled 92 (54.44%) were females and the mean age of the participants was 37.2 (SD 12.8) years. The average age of onset of mental illness was 24.68 (SD 8.87) years. Overall the quality of life was poor with 113 (66.86%) of the participants having a poor physical component summary of the quality of life, while 137 (81.07%) reporting a poor mental component summary of the quality of life (Table 1).

**Table 1:** Sociodemographic characteristics of the participants (N = 169)

Characteristic	Mean (SD) OR n (%)
Age	37.23 (12.83)
The average number of episodes in a year	01 (2.35)
Time since the last episode (months)	15.55 (27.65)
Age at onset of illness	24.6815.55 (8.8727.65)
Number of admissions in the previous year	01.02 (1.95)
*Sex*	
Female	92 (54.44)
Male	77 (45.56)
*Religion*	
Catholic	72 (42.60)
Protestant	66 (39.05)
Muslim	12 (7.10)
Others	19 (11.24)
*Employment status*	
Subsistence farmer	59 (34.91)
Business	39 (23.08)
Civil servant	22 (13.02)
Not employed	42 (24.85)
Others	7 (4.14)
*Area of residence*	
Urban	47 (27.81)
Rural	122 (72.19)
*Level of education*	
No formal education	13 (7.69)
Some primary	29 (17.16)
Completed primary	17 (10.06)
Some secondary	46 (27.22)
Completed secondary	23 (13.61)
Tertiary	41 (24.26)
*Marital status*	
Single	66 (39.05)
Married	63 (37.28)
Separated	30 (17.75)
Widowed	10 (5.92)
*HIV status*	
Negative	128 (75.74)
Positive	19 (11.24)
Unknown	22 (13.02)
*History of suicide ideation*	
Yes	61 (36.09)
No	108 (63.91)
*History of suicide attempt*	
Yes	44 (26.04)
No	125 (73.96)
*History of psychotic symptoms*	
Yes	89 (52.66)
No	80 (47.34)
*Physical quality of life*	
Poor	113 (66.86)
Good	56 (33.13)
*Mental quality of life*	
Poor	137 (81.07)
Good	32 (18.93)

The overall mean subscale scores of the study sample were below 82 with the highest attained value being 81 which was in the category of bodily pain. Most of the parameters scored between the 50th and 60th percentiles. Table 2 shows the mean and standard deviation of the sub-scales, physical component summary and mental component summary of the entire sample aggregated by sex. The standardized physical component score and the mental component summary were all below average 45.06 (SD 8.44) and 41.95 (SD 8.45) respectively. There were minor differences between the sub-scale means between men and women. Women scored less than males in mental health, social functioning, vitality, bodily pain and general health hence the lower score for the overall mental component summary.

**Table 2:** SF-36 subscale mean scores stratified by sex

Scales	Total sample Mean (SD)	Male Mean (SD)	Female Mean (SD)
Physical function	79.47 (21.75)	79.16 (24.41)	79.73 (19.39)
Physical role limitation	56.36 (45.48)	53.57 (47.59)	58.70 (43.76)
Emotional role limitation	55.23 (46.30)	54.98 (46.44)	55.43 (46.43)
Vitality	59.97 (19.51)	61.56 (19.66)	58.64 (19.39)
Mental health	69.18 (18.74)	70.81 (17.83)	67.83 (19.47)
Social function	73.45 (24.18)	74.19 (24.02)	72.83 (24.42)
Bodily pain	81.05 (25.70)	83.05 (21.71)	79.38 (28.63)
General Health	61.04 (21.32)	62.08 (20.09)	60.16 (22.37)
*Summary score*			
Physical component summary (PCS	45.06 (8.44)	45.13 (8.36)	45.01 (8.55)
Mental component summary (MCS0	41.95 (8.45)	42.45 (8.53)	41.53 (8.40)

On bivariate analysis we run sociodemographic and clinical factors against mental and physical quality of life as our outcome variables. History of suicidal thoughts and psychosis were significantly associated with both poor physical and mental quality of life (Table 3).

**Table 3:** Bivariate analysis of the factors and poor physical quality of life (after adjusting for age and sex)

Characteristic	Adjusted OR, 95% CI *P* value	Adjusted OR, 95% CI *P* value
	Mental quality of life	Physical quality of life
Residence (rural)	0.98 (0.41–2.31),0.96	0.57 (0.28–1.14), 0.11
Employment status	0.87 (0.64–1.18), 0.38	1.11 (0.85–1.44), 0.46
Level of education	0.85 (0.67–1.09), 0.20	1.00 (0.82–1.23), 0.97
Marital status	1.37 (0.82–2.29), 0.23	0.93 (0.61–1.42), 0.74
HIV status (positive)	0.77 (0.42–1.41), 0.39	1.14 (0.72–1.80), 0.56
Age at onset of illness	1.01 (0.96–1.07), 0.59	1.14 (0.72–1.80), 0.56
Number of episodes/year	0.80 (0.59–1.08), 0.14	0.89 (0.75–1.07), 0.24
Time since last episode	0.99 (0.98–1.01), 0.54	1.01 (0.99–1.02), 0.147
Admissions in the last year	0.95 (0.76–1.19), 0.65	0.86 (0.69–1.06), 0.17
Suicidal thoughts	2.98 (1.13–7.81), 0.03	1.99 (0.97–4.08), 0.06
Suicidal attempt	1.05 (0.42–2.61), 0.91	0.74 (0.36–1.54), 0.42
Psychotic symptoms	2.56 (1.13–5.77), 0.02	1.819 (0.958–3.4566), 0.026

At multivariable logistic regression analysis, poor physical quality of life had a statistically significant association with suicidal thoughts [AOR 2.75 (1.14–6.63), *P* = 0.02], while poor mental quality of life had a statistically significant association with history of psychosis [AOR 3.94 (1.22–12.71) *P* = 0.02] and suicidal thoughts [AOR2.46 (1.07–5.64), *P* = 0.03] (Table 4).

**Table 4:** Multivariable model depicting the poor quality of life (mental and physical quality of life)

Characteristic	Adjusted OR, 95% CI *P* value	Adjusted OR, 95% CI *P* value
	Poor mental quality of life	Poor physical quality of life
Time since last episode	0.99 (0.97–1.01), 0.41	1.01 (0.99–1.02), 0.2174
Average number of episode in a year	0.79 (0.58–1.09), 0.15	0.89 (0.75–1.08) 0.25
Suicidal thoughts	3.94 (1.22–12.71), 0.02	2.75 (1.14–6.63), 0.02
Suicide attempts	0.50 (0.16–1.53), 0.23	1.64 (0.84–0.83)0.07
Psychotic symptoms	2.46 (1.07–5.64), 0.03	N/A1.89 (0.98–3.66) 0.06
Admissions in the previous year	−	0.84 (0.67–1.06), 0.12

## Discussion

Results from our study show that bipolar disorder is associated with impairment in the health-related quality of life with impairment in both physical and mental aspects of life. This finding is consistent with other studies in both low and high-income countries in which bipolar disorder was found to impair the quality of life of patients with the disorder [29, 56, 57]. In the study by Kebede et al. presence of depressive symptoms in the most recent episode was associated with poorer quality of life [56]. Sixty-seven percent (67%) of the participants in our study had a poor physical quality of life, whereas 81% had a poor mental quality of life which is in agreement with previous studies that the physical and mental components of life tend to be lower among people with bipolar disorder compared to the general population [58]. However, the mental quality of life was more affected compared to the physical aspect among our sample and this may be explained by the fact that bipolar disorder affects different psychological domains including self-esteem, thinking, memory, and concentration compared to the physical domains [59, 60]. Additionally, the worry about living with a chronic recurring illness may contribute to worsened mental quality of life [60].

Our study found that history of having had suicidal thoughts and psychotic symptoms in the most recent episode were associated with poor physical and mental quality of life. These findings are in agreement with previous studies indicating that suicidal ideations are associated with poor quality of life [19, 61]. The association between suicidal ideations and poor quality of life among people with bipolar disorder has been linked to challenges of life satisfaction among people with mental health problems [62]. It has also been documented that on average people with bipolar disorder spend more time depressed than manic which may also explain the link between suicidal ideations and worsened quality of life [63]. The association between psychotic symptoms and quality of life has been reported in previous studies [64–66]. The association between psychotic symptoms and poor quality of life among people with bipolar disorder may be partly due to delayed response to treatment of the psychotic symptoms which affects functional recovery [67]. In addition, psychosis during manic episodes has been associated with longer periods of illness, higher relapse rates and increased symptom severity [25, 26, 65]. Moreover, the presence of psychotic symptoms during manic episodes are considered as a sign of poor prognosis [64].

Our study did not register any significant difference between the physical component summary scores between men and women. This is inconsistent with findings from a study by De la Cruz in which women had a lower physical summary score than men [68]. However in the study by de la Cruz the reported lower physical quality of life among women compared to men in their sample was linked to high risk of medical comorbidities among women compared to men [68]. In our study we did not assess for medical comorbidities and this may explain the difference in our findings. However, the mental component summary in our study was lower among women than men. The low mental component summary among women in our study may be linked to the overwhelming emotional and environmental stigma imparted on women in the Ugandan setting compared to men [69]. The lower mental component summary (MCS) in regards to the gender is in agreement with what has been reported in previous studies [68]. Female sex has been linked to poorer quality of life among patients with bipolar disorder due to the fact that women with bipolar disorder tend to suffer more depressive episodes compared to men yet depressive symptoms are associated with more impairment in the quality of life among patients with bipolar disorder [23, 24, 70],

Contrary to what has been reported in some studies [71–73], we did not find statistically significant associations between number of episodes or number of admissions in the past year with quality of life. However, our findings are in agreement with findings by Sierra et al. [29] who found that duration of mental illness and number of previous episodes did not predict quality among patients with bipolar disorder. Studies that have found the relationship between number of episodes and impaired quality of life have pointed out that depressive episodes tend to have a greater impact on quality of life than the manic episodes [13]. In addition, studies that have found significant associations between number of episodes or number of admissions with quality of life among patients with bipolar disorder show a higher number of manic and depressive episodes and several admissions in the past [73]. In our study our participants had on average one episode and one admission in the previous year which may explain why number of episodes and admissions in the previous year did not predict quality of life.

Our study had some limitations. First, we collected data from participants who were receiving regular mental health services from a well-structured mental health clinic and hence their views may not be a true representation of all the patients with BD in rural southwestern Uganda. Secondly, we were asking participants about events that took place in the past and this could have resulted in recall bias hence inconsistent responses as participants reported past events. Third, we did not use standard scales to confirm the diagnosis of the participants and as such we could have enrolled some participants who did not have a true diagnosis of bipolar disorder. However, since the clinic is run by qualified psychiatrists we are confident that the findings reflected in this study in relation to quality of life represent those of patients with bipolar disorder in our setting.

## Conclusions

Our findings indicate that patients with bipolar disorder have a poor quality of life and that having had suicidal thoughts and psychosis predict poor quality of life among patients with bipolar disorder. Health care providers must bear in mind the impact bipolar disorder has on quality of life of those who suffer this condition so that appropriate measures are put in place to improve health outcomes of the patients. There is need to pay close attention to the psychotic symptoms and suicidal thoughts during the management of patients with bipolar disorder so that these can be controlled in addition to mood stabilization to improve physical and mental health outcomes. Additionally, the management of patients with bipolar disorder should focus on both the physical and mental health domains to improve quality of life.

## Data Availability

The dataset (s) supporting the conclusions of this article is (are) included within the article (and its additional file(s)).

## Abbreviations

BD: Bipolar disorder
YLL: Years of life lost
YLD: Years lived with disability
QoL: Quality of life
MRRH: Mbarara Regional Referral Hospital
REC: Research Ethics Committee
PCS: Physical comment summary
MCS: Mental component summary
SF-36: Health survey short form-36
